# Health-Associated Nutrition and Exercise Behaviors in Relation to Metabolic Risk Factors Stratified by Body Mass Index

**DOI:** 10.3390/ijerph16050869

**Published:** 2019-03-09

**Authors:** Jui-Hua Huang, Ren-Hau Li, Shu-Ling Huang, Hon-Ke Sia, Wei-Ting Hsu, Feng-Cheng Tang

**Affiliations:** 1 Department of Golden-Ager Industry Management, Chaoyang University of Technology, Taichung 413, Taiwan; Juihua55@ms35.hinet.net; 2 Occupational Health Center, Changhua Christian Hospital, Changhua 500, Taiwan; 3 Department of Psychology, Chung Shan Medical University, Taichung 402, Taiwan; davidrhlee@yahoo.com.tw (R.-H.L.); shuling@csmu.edu.tw (S.-L.H.); 4 Room of Clinical Psychology, Chung Shan Medical University Hospital, Taichung 402, Taiwan; 5 Division of Endocrinology and Metabolism, Changhua Christian Hospital, Changhua 500, Taiwan; 90279@cch.org.tw; 6 Department of Construction Engineering, Chaoyang University of Technology, Taichung 413, Taiwan; wthsu@cyut.edu.tw; 7 Department of Leisure Services Management, Chaoyang University of Technology, Taichung 413, Taiwan; 8 Department of Occupational Medicine, Changhua Christian Hospital, Changhua 500, Taiwan; 9 School of Medicine, Kaohsiung Medical University, Kaohsiung 807, Taiwan

**Keywords:** nutrition, exercise, metabolic risk factors, health promotion, worker

## Abstract

This study aimed to investigate the relationships of nutrition and exercise behaviors on metabolic risk factors (MRF) when body mass index (BMI) was considered. Health-associated nutrition and exercise behaviors were assessed by a questionnaire, anthropometric values, blood pressure and biochemical determinations that were obtained from 4017 workers. The nutrition score was negatively associated with triglycerides in the overweight subgroup and with systolic blood pressure (SBP) in the obese subgroup. The exercise score was negatively associated with triglycerides and waist circumference (WC) and positively associated with SBP and high-density lipoprotein cholesterol (HDL-C) in the ideal weight subgroup as well as being negatively associated with WC and positively associated with HDL-C in the overweight subgroup. Similarly, the exercise score was negatively associated with WC and positively associated with SBP in the obese subgroup. However, no significant association was found between nutrition or exercise behavior and MRF in the underweight subgroup. In conclusion, the relationships of exercise and nutrition behaviors on MRF varied for different levels of BMI. Exercise showed a significant association with lower WC. Moreover, its effect showed a gradient trend in accordance with the levels of BMI. For ameliorating MRF, exercise seemed to have better effects than nutrition behavior, especially in the ideal weight subgroup.

## 1. Introduction

Metabolic risk factors (MRFs) such as abdominal obesity, dyslipidemia, hyperglycemia, and hypertension are causally linked to the risk of cardiovascular disease (CVD) [1,2]. CVD is a major cause of death in Taiwan and in other countries worldwide [3,4]. Heart disease, cerebrovascular diseases, diabetes, and hypertension held the second, fourth, fifth, and eighth positions, respectively, among the 10 leading causes of mortality in 2016 in Taiwan [4]. In causes of death in Taiwan’s male workers, heart disease, cerebrovascular diseases, and diabetes ranked second, fifth, and sixth, respectively [5]. In addition, diabetes, cerebrovascular disease, and heart disease were the three leading causes of death in female workers [5]. Several healthy behaviors such as exercising and eating a well-balanced diet may reduce the risk of MRFs, whereas unhealthy behaviors such as smoking and excessive drinking may increase the risk of MRFs [6,7,8]. Therefore, helping workers maintain healthy behaviors is an important strategy for reducing MRFs in worksite health promotion [9,10].

Nutrition behavior is associated with CVD-related MRFs [11,12]. Evidence suggests that healthy nutrition behavior such as a diet high in fruits and vegetables and fish may lower the risk of CVD and death [13]. A diet low in saturated fat and trans fats may reduce CVD risk factors such as high blood pressure (BP) and an unfavorable blood lipid level [14,15,16]. Conversely, unhealthy nutrition behavior such as excessive salt intake is associated with inadequate BP control and overall cardiovascular risk [17,18]. High sugar intake has adverse effects on BP and blood lipids [19,20], and may raise the risk of diabetes [21]. There is also evidence that frequently eating high-energy foods such as processed foods that are high in fats and sugars can also cause obesity and raise cardiovascular risk [12,18]. Therefore, according to the above-mentioned evidence, important CVD-related MRFs may be modifiable by changing nutrition behavior.

Exercise behavior is also one of the components important to managing weight, reducing MRFs, and maintaining cardiovascular health [22,23]. Several studies have reported that regular and appropriate exercise showed a positive effect on BP, weight control, and blood lipids in adults [22,24]. Other studies have also reported that appropriate physical exercise can reduce CVD-related MRF in patients with diabetes [23,25]. Conversely, low levels of physical exercise may raise the development of MRFs and the risk of CVD mortality. To enhance the benefits of exercise on health, the general suggestion is that individuals should do 20 to 60 min of exercise three to five days per week with an exercise intensity at 50 to 85 percent of the maximum heart rate [26]. To improve overall cardiovascular health and lower blood pressure and unfavorable cholesterol, the American Heart Association recommends that adults perform moderate to vigorous intensity aerobic activity three or four days per week [27]. 

The BMI is a commonly applied index of weight categories and is used as a predictor of obesity-related health risk [28]. Increased BMI has been found to be associated with an increased risk of morbidity and mortality from CVD in several populations [29]. Individuals with a higher BMI may have an excess propensity toward adipose tissue when compared to individuals with ideal BMI [29]. Adipokines released from adipose tissue may induce insulin resistance, endothelial dysfunction, hypercoagulability, and inflammation, all of which can develop into CVD [29]. A study has also shown that higher BMI is associated with microvascular endothelial dysfunction in patients with suspected coronary artery disease [30]. If people with a high BMI adopt an unhealthy lifestyle such as unhealthy dietary patterns and sedentary behaviors, they may be presumed to have a higher metabolic- and CVD-related risk [6,7,12]. The advantage of eating healthily and exercising appropriately with respect to reducing MRFs has been observed in obese individuals [31,32,33]. It is unclear whether this advantage is the same for individuals with other levels of BMI. Existing studies clarifying the relationships of nutrition and exercise with lower CVD-related MRFs for each level of body mass index (BMI) are especially scant. Among these limited studies, BMI levels were classified into merely two or three subgroups [34,35]. A comprehensive understanding of the role of BMI level in the relationships between healthy behaviors and MRFs is needed. In addition, workers are a high-risk population for metabolic- and CVD-related risk [5]. The effects of healthy behaviors on the overall CVD risk using the Framingham risk score were explored in our previous study [36]. In this study, we aimed to investigate the relationships of nutrition and exercise behaviors on MRFs when four BMI levels were stratified.

## 2. Materials and Methods 

### 2.1. Ethics Statement

This study was one component of the Taiwan Workplace Health-Promotion Scheme. The study was conducted according to the Declaration of Helsinki and was approved by the Institutional Review Board of the Changhua Christian Hospital in Taiwan with a waiver of informed consent (CCHIRB No: 120606).

### 2.2. Study Population

This cross-sectional study was conducted in 2012 by the Center for Occupational Health. The workers were recruited via convenience sampling from four manufacturing companies in central Taiwan. The workers’ occupations consisted of management, white-collar workers (including professionals, technicians, office workers, and service workers), and blue-collar workers (including crafts worker and machine operators) in the present study. The principal activities of these workers included manufacturing electronic components, pumps, motor vehicle parts, and transport equipment. A total of 5096 workers 20 years of age or older were invited to complete a questionnaire where they self-reported their personal information, health-associated nutrition and exercise behaviors, and occupation. Data on MRFs were collected through the contacted companies’ annual health screening required by Taiwan regulations. After excluding 1079 workers lacking the necessary information on personal data, nutrition and exercise health behaviors, or MRFs, 4017 workers consisting of 3286 male workers and 731 female workers were included in the final analysis.

### 2.3. Assessment of Nutrition and Exercise Behaviors

The Health-Promoting Lifestyle Profile II (HPLP II) [37] is a modified version of the Health-Promoting Lifestyle Profile [38] that has been used extensively in research and is reported to have sufficient validity and reliability for use in various populations [39,40,41]. It composes of six subscales including nutrition, physical activity, health responsibility, stress management, interpersonal relations, and spiritual growth. For research purposes, only nutrition and physical activities subscales were adopted in the present study to assess the nutrition and exercise behaviors. Nutrition behavior was evaluated using nine items including (1) Choosing a diet low in fat, saturated fat, and cholesterol; (2) Limiting the use of sugars and sweets; (3) Eating daily servings of bread, cereal, rice, and pasta; (4) Eating daily servings of fruit; (5) Eating daily servings of vegetables; (6) Eating daily servings of meat, poultry, fish, dried beans, eggs, and nuts; (7) Eating daily servings of milk, yogurt, or cheese; (8) Reading labels to identify nutrients; and (9) Eating breakfast. For information on the recommended number of daily servings for each food group, participants were referred to the Taiwanese dietary guidelines. In addition, eight items for exercise health behavior were (1) Following a planned exercise program; (2) Exercising vigorously for 20 or more minutes at least three times per week; (3) Taking part in light to moderate physical activity; (4) Taking part in leisure-time (recreational) physical activities; (5) Doing stretching exercises at least three times per week; (6) Getting exercise during usual daily activities; (7) Checking pulse rate when exercising; and (8) Reaching the target heart rate when exercising. Items were scored as Never = 1, Sometimes = 2, Often = 3, and Routinely = 4. A score for nutrition or exercise behavior was obtained by calculating the mean of the responses to subscale items. A higher mean score was associated with a greater level of participation in healthy nutrition or exercise behavior. In this study, the subscales of nutrition and exercise behaviors for the Taiwanese version of the HPLP II revealed an acceptable internal consistency, with Cronbach’s alphas of 0.85 and 0.78, respectively.

### 2.4. Definition of Different BMI Levels

Anthropometric measurements included height and weight. The calculation for BMI is weight in kilograms divided by height in meters squared [(kg)/(m^2^)]. BMI status was categorized as underweight (BMI < 18.5), ideal weight (18.5 ≤ BMI < 24.0), overweight (24.0 ≤ BMI < 27.0), and obese (BMI ≥ 27.0), according to the definition of the Health Promotion Administration, Ministry of Health and Welfare in Taiwan [42].

### 2.5. Measurements of MRFs

The measurement of MRFs included WC, systolic blood pressure (SBP) and/or diastolic blood pressure (DBP), fasting glucose level (FBG), triglycerides (TG), and HDL-cholesterol (HDL-C) [43,44]. The measurement of WC (cm) was performed by trained health personnel in accordance with the International Standards for Anthropometry and Kinesiology (ISAK) [45]. The BP measurement was taken using a validated digital sphygmomanometer (HEM-7310, Omron, Kyoto, Japan) while the participant was in a seated position after at least five minutes of rest. In addition, blood samples were collected after at least eight hours of overnight fasting, and the medical laboratory (certified ISO 15189) measured the biochemical parameters using a biochemical auto-analyzer (TBA-200FR, Toshiba, Tokyo, Japan). FBG was analyzed using an enzymatic UV test (hexokinase method). TG was measured using a series of coupled enzymatic reactions. HDL-C was measured by direct methods.

### 2.6. Statistical Analysis

All statistical procedures were performed using SPSS 17.0 statistical software (SPSS Inc., Chicago, IL, USA). For categorical variables in the contingency table, data were presented in number (n) and percent (%) and were analyzed by the Chi-square test. Continuous variables were analyzed by one-way ANOVA (>2 groups) tests followed by Scheffe’s post-hoc multiple comparisons. Continuous data were presented in mean ± SD with median. The bivariate correlation test was used to calculate the crude correlations of healthy behaviors and individual items with MRFs. Multiple linear regression analysis with adjustments for gender and age was applied to determine the relationships between healthy behaviors and MRFs, stratified by BMI level. Data were presented in unstandardized coefficients (B) and standardized coefficients (β). A *p*-value of less than 0.05 was considered statistically significant. 

## 3. Results

### 3.1. Descriptive Statistics of the Participants’ Personal Characteristics by BMI Levels

Table 1 shows the participants’ personal characteristics. Of the 4017 workers, 112 (2.8%) were classified as underweight, 1852 (46.1%) were classified as ideal weight, 1287 (32.0%) were classified as overweight, and 766 (19.1%) were classified as obese. BMI levels were significantly associated with gender, age, and health-associated nutrition and exercise behaviors.

### 3.2. Relationships between BMI Levels and Metabolic Risk Factors

Table 2 shows the relationships between BMI levels and MRFs. The mean of each MRF in the four different BMI subgroups among workers was significantly different (*p* < 0.001). Individuals with a higher BMI may have a significant propensity toward the mean of each MRF when compared to lower-BMI individuals. In Scheffe’s post-hoc comparison, WC, TG, and HDL-C showed a salient trend (*p* < 0.001); the data were worse along with higher BMI subgroups. For FBG, SBP, and DBP, all of the differences between any two subgroups were also significant except those between the ideal weight subgroup and the underweight subgroup.

### 3.3. Crude Correlations of Metabolic Risk Factors and Health Behavior

Table 3 shows the crude correlations of the MRFs with nutrition and exercise health behaviors for all of the participants as a whole and for each stratification by the BMI level. For the underweight subgroup, nutrition behavior showed a significantly negative correlation with WC; exercise behavior did not show significant correlation with any of the MRFs. For the ideal weight subgroup, nutrition behavior showed a significantly negative correlation with WC and positive correlation with DBP and HDL-C; and exercise behavior showed a significantly negative correlation with TG and positive correlations with SBP, DBP, and HDL-C. For the overweight subgroup, nutrition behavior showed a significantly negative correlation with TG and positive correlations with SBP, DBP, and HDL-C; and exercise behavior was positively correlated with SBP, DBP, and HDL-C. For the obesity subgroup, nutrition behavior showed a significantly positive correlation with DBP; exercise behavior showed a significantly negative correlation with WC and positive correlations with SBP and DBP. Additionally, the crude correlations of each item of nutrition and exercise behaviors and MRFs for all of the participants as a whole and for each stratification by BMI level are presented in the Supplementary Materials.

### 3.4. Metabolic Risk Factors in Relation to Nutrition and Exercise Health Behavior According to Different BMI Levels

Using multiple linear regression analysis with adjustments for gender and age, the relationships of nutrition and exercise behavior on the MRFs are presented by the different BMI levels shown in Table 4. Results from the ideal weight subgroup showed that nutrition behavior entailed a negative correlation of SBP, but the statistical significance was not shown. For the overweight subgroup, nutrition behavior was significantly negatively correlated with TG. For the obese subgroup, nutrition behavior was significantly negatively correlated with SBP. In addition, exercise behavior was significantly negatively correlated with WC and TG, and positively correlated with SBP and HDL-C in the ideal weight subgroup. For the overweight subgroup, exercise behavior showed a significantly negative correlation with WC and a positive correlation with HDL-C. For the obese subgroup, exercise behavior showed a significantly negative correlation with WC and a positive correlation with SBP. However, no significant relationship was found between the MRFs and nutrition or exercise behavior for the underweight subgroup.

## 4. Discussion

This study aimed to investigate the relationships of nutrition and exercise behaviors on MRFs when four different BMI levels were stratified. Our data suggested that (1) for underweight workers, healthy nutrition and exercise behaviors were not significantly associated with any of the MRFs; (2) for ideal weight workers, exercise was associated with a lower level of WC and TG as well as a higher level of HDL-C, whereas exercise was associated with a higher level of SBP; (3) for overweight workers, healthy nutrition behavior was associated with lower TG, and exercise was associated with lower WC and higher HDL-C; and (4) for obese workers, healthy nutrition behavior was associated with lower SBP, whereas exercise was associated with higher SBP. Additionally, exercise was associated with lower WC.

A healthy diet is high in fruits, vegetables, whole grains, lean meats, and fiber, and low in saturated fats, trans fats, cholesterol, sodium, and added sugars [46,47]. Previous studies have suggested that healthy nutrition behavior has the benefit of reducing the probability of metabolic abnormalities and preventing CVD [11,12]. Nevertheless, the relationships between healthy nutrition behavior and MRFs were not apparent when the present study was further classified by BMI. This study only found that nutrition behavior was associated with lower TG in the overweight subgroup and lower SBP in the obese subgroup. As for the underweight or ideal weight subgroup, healthy nutrition behavior did not show any significant relationship with the MRFs. The possible reason for this is that the global nutrition behavior score adopted in the present study might be different from the actual food consumption used in some other studies. The exact differences between these two measurements need to be evaluated in further studies.

Regular and effective exercise has shown a positive effect on weight control, blood glucose, blood lipids, and BP [22,24]. This study found differences in the effects of healthy exercise behavior on MRFs when stratified by BMI level. Our data showed that exercise behavior was associated with lower WC and TG as well as higher HDL-C, but higher SBP in the ideal weight subgroup. In particular, exercise had a salient effect on reducing WC for three subgroups (ideal weight, overweight, and obese). In addition, this effect showed a gradient trend in accordance with the level of BMI. It is known that physical activity can increase caloric expenditure. Energy consumption through long-term exercise is mainly due to free fatty acids being released from the decomposition of adipose tissue [48]. Therefore, extra energy is not stored as adipose tissue and contributes to a reduction in body fat mass and WC [24,49]. In general, the amount of adipose tissue is highest for obese individuals, followed by overweight individuals, and those of ideal weight. Therefore, the effect of exercise on a reduction in WC would be the most significant for obese individuals, followed by overweight individuals, and those of ideal weight. However, the obese workers needing to exercise more reported a lower level of exercise health behavior than those of ideal body weight or those who were overweight. The health-promoting strategy in the workplace therefore should be targeted to strengthen the exercise behavior of obese individuals.

In this study, exercise behavior was associated with increased HDL-C and decreased TG in the ideal weight subgroup and with increased HDL-C in the overweight subgroup. Several studies have confirmed that exercise can help individuals improve blood lipid levels and keep their cardiovascular system healthy [22,23,50]. Healthy exercise had a noticeable effect on blood lipids in the ideal weight subgroup, but the effect was not significant in the obese subgroup. This could be because obese individuals have a higher propensity toward highly sensitive C reactive protein (hsCRP, inflammation biomarker) compared to ideal weight individuals [51]. The higher level of inflammation may predict a higher risk of developing metabolic abnormalities including abnormal levels of blood lipids [52]. Exercise behavior could be insufficient for obese workers to improve their blood lipid levels. Obese workers may still need other methods such as anti-inflammatory drugs to ameliorate their blood lipids [29].

Healthy exercise behavior has the benefit of reducing BP whereas sedentary behaviors are linked to high blood pressure [53,54]. In general, exercise may cause BP to rise for a short time and drop back to normal when the exercise stops [55]. However, this study found that exercise was associated with raised SBP in the ideal weight and obese subgroups. The possible explanation for the raised SBP in the present study is that the spot-check measurement of BP did not reflect its long-term status even though efforts were made to achieve accuracy. Furthermore, in addition to nutrition and exercise behaviors, BP may be susceptible to many factors such as stress, smoking, some diseases, medication, and results from a complex interaction of genes and environmental factors [56,57]. Workers with higher BP should regularly check their BP and consult their physician for necessary treatment. In addition, further research that clarifies other possible reasons and mechanisms for the positive relationship between higher SBP and exercise behavior in ideal weight and obese subgroups is recommended. In terms of the relationship between healthy behaviors and MRF in the underweight subgroup, no statistically significant results were observed. Due to low free-fat mass and low fat mass, underweight individuals generally have a low basal metabolic rate (BMR) [58]. A low BMR can lead to low metabolic function and less fat reduction. This may be the reason for the insignificant effects of healthy behaviors on WC and other MRFs in the underweight subgroup. Nevertheless, underweight individuals have been found to be associated with a high risk of health-related problems such as osteoporosis, infection, and decreased immune function [59,60]. Therefore, the needs of workplace health promotion for underweight individuals should be targeted in different ways.

The present study showed that the relationships of exercise and nutrition behaviors on MRF varied for different levels of BMI. The findings in this study bring about some policy implications for employee health promotion. Workplace health promotion practitioners should consider the BMI levels of workers when nutrition and exercise behaviors are adopted as strategies with the aim of preventing MRFs. Although this study shed light on the relationships of nutrition and exercise behaviors on MRF classified by the level of BMI, this study had several limitations. First, it was a cross-sectional study and could not establish the causal direction among the research variables. Second, the assessments of the nutrition and exercise behaviors were highly dependent on the self-reported questionnaire. Therefore, biased results may have occurred when participants overestimated or underestimated their own health behaviors. Third, BMI can be applied to screen for weight categories and used as a predictor of fat-related health risk. However, BMI is not a strong predictor of body fat in participants who have normal BMI but high body fat. Therefore, combining BMI with other methods for assessing body fat in further research is recommended. Fourth, the participants’ physical work load, smoking, and alcohol consumption were not included in the measurement. However, these factors may confound the association between exercise and nutrition behaviors with MRFs. The results should therefore be interpreted with caution.

## 5. Conclusions

The relationships of health-associated nutrition and exercise behaviors on MRFs were not found to be the same for workers with different BMI levels. Exercise had a salient effect on reducing WC, and this effect showed a gradient trend for the BMI level. For ameliorating other metabolic risk factors besides WC, exercise seemed to have more obvious effects than nutrition behavior, especially in the ideal weight subgroup. Therefore, it is suggested that workplace health promotion personnel should develop relevant strategies to meet the needs of workers with different BMI levels.

## Figures and Tables

**Table 1:** Descriptive statistics of the participants’ personal characteristics by BMI levels *.

Variables ^†^	Total	BMI (kg/m^2^) Levels				*p*
		BMI < 18.5 (Underweight)	18.5 ≤ BMI < 24 (Ideal Weight)	24 ≤ BMI < 27 (Overweight)	BMI ≥ 27 (Obesity)	
*n* (%)	4017 (100)	112 (2.8)	1852 (46.1)	1287 (32.0)	766 (19.1)	
Gender						
Male	3286 (81.8)	68 (2.1)	1412 (43.0)	1121 (34.1)	685 (20.8)	<0.001
Female	731 (18.2)	44 (6.0)	440 (60.2)	166 (22.7)	81 (11.1)	
Age (year)	43.1 ± 10.0	41.5 ± 10.6	43.2 ± 10.1	44.0 ± 9.8	41.8 ± 9.6	<0.001
Nutrition health behavior	2.47 ± 0.44	2.51 ± 0.49	2.49 ± 0.45	2.46 ± 0.44	2.43 ± 0.40	0.007
Exercise health behavior	1.96 ± 0.56	1.90 ± 0.47	1.98 ± 0.58	1.96 ± 0.56	1.91 ± 0. 53	0.015

* Continuous data are presented in mean ± SD. ^†^ Categorical data are presented in number (*n*) and percent (%). *p*-value indicates significant level.

**Table 2:** Relationships between the BMI levels and metabolic risk factors ^†^.

Variables	WC (cm)	FBG (mg/dL)	SBP (mmHg)	DBP (mmHg)	HDL-C (mg/dL)	TG (mg/dL)
BMI (kg/m^2^) levels						
BMI < 18.5 (Underweight, UW)	66.5 ± 5.2 [66.0]	88.0 ± 10.2 [87.0]	115.9 ± 15.7 [114.0]	75.3 ± 11.0 [75.0]	63.8 ± 12.0 [63.2]	79.5 ± 34.0 [74.0]
18.5 ≤ BMI < 24 (Ideal weight, IW)	76.3 ± 6.2 [76.0]	91.0 ± 16.2 [89.0]	119.5 ± 14.4 [119.0]	76.3 ± 10.2 [76.0]	56.5 ± 12.9 [55.0]	111.5 ± 81.6 [93.0]
24 ≤ BMI < 27 (Overweight, OW)	84.7 ± 5.4 [85.0]	94.5 ± 19.6 [92.0]	124.7 ± 14.6 [124.0]	79.7 ± 11.0 [80.0]	50.1 ± 11.0 [48.5]	147.7 ± 105.5 [123.0]
BMI ≥ 27 (Obesity, OB)	93.5 ± 8.0 [93.0]	97.9 ± 22.8 [93.0]	130.1 ± 16.7 [129.0]	83.4 ± 12.5 [83.0]	46.3 ± 10.0 [45.9]	172.0 ± 123.7 [142.0]
F	1646.353	29.415	104.313	84.209	191.023	91.122
*p*	<0.001	<0.001	<0.001	<0.001	<0.001	<0.001
Scheffe’s post-hoc comparison	OB > OW > IW > UW	OB > OW > UW OB > OW > IW	OB > OW > UW OB > OW > IW	OB > OW > UW OB > OW > IW	UW > IW > OW > OB	OB > OW > IW > UW

^†^ Relationships between BMI levels and metabolic risk factors were examined by one way ANOVA F-tests with *p*-value significance level, followed by Scheffe’s post-hoc multiple comparisons. Data are presented in mean ± SD and [median]. Abbreviations: WC, Waist circumference; FBG, Fasting blood glucose; SBP, Systolic blood pressure; DBP, Diastolic blood pressure; TG, Triglyceride; HDL-C, High-density lipoprotein cholesterol; BMI, body mass index.

**Table 3:** Crude correlations of metabolic risk factors and nutrition and exercise health behavior ^†^.

Variables	WC (cm)		FBG (mg/dL)		SBP (mmHg)		DBP (mmHg)		HDL-C (mg/dL)		TG (mg/dL)	
	r	*p*	r	*p*	r	*p*	r	*p*	r	*p*	r	*p*
Nutrition	−0.075	<0.001	−0.005	0.763	0.013	0.425	0.069	<0.001	0.098	<0.001	−0.043	0.007
Exercise	−0.054	0.001	−0.015	0.331	0.086	<0.001	0.098	<0.001	0.092	<0.001	−0.043	0.007
Different BMI levels												
BMI < 18.5 (Underweight)												
Nutrition health behavior	−0.186	0.049	0.139	0.143	0.088	0.354	0.102	0.286	0.116	0.223	0.159	0.095
Exercise health behavior	−0.143	0.132	0.047	0.620	0.071	0.459	0.045	0.636	0.078	0.415	0.015	0.872
18.5 ≤ BMI < 24 (Ideal weight)												
Nutrition health behavior	−0.077	0.001	−0.002	0.947	−0.005	0.843	0.061	0.008	0.094	<0.001	−0.019	0.410
Exercise health behavior	−0.018	0.427	0.001	0.957	0.099 ^†^	<0.001	0.110	<0.001	0.072	0.002	−0.054	0.021
24 ≤ BMI < 27 (Overweight)												
Nutrition health behavior	−0.035	0.207	−0.002	0.956	0.069	0.013	0.113	<0.001	0.084	0.003	−0.057	0.040
Exercise health behavior	−0.036	0.199	−0.015	0.594	0.086	0.002	0.102	<0.001	0.121	<0.001	−0.019	0.506
BMI ≥ 27 (Obesity)												
Nutrition health behavior	−0.007	0.851	0.010	0.790	0.031	0.389	0.091	0.011	0.047	0.199	−0.021	0.570
Exercise health behavior	−0.077	0.034	−0.027	0.458	0.132	<0.001	0.144	<0.001	0.065	0.074	−0.032	0.370

^†^ Correlations of metabolic risk factors and nutrition and exercise health behavior were examined by bivariate correlation test with *p*-value significance level. Abbreviations: WC, Waist circumference; FBG, Fasting blood glucose; SBP, Systolic blood pressure; DBP, Diastolic blood pressure; TG, Triglyceride; HDL-C, High-density lipoprotein cholesterol; BMI, body mass index.

**Table 4:** Linear regression models predicting metabolic risk factors in relation to nutrition and exercise health behavior according to different BMI levels ^‡^.

Variables	WC (cm)			FBG (mg/dL)			SBP (mmHg)			DBP (mmHg)			HDL-C (mg/dL)			TG (mg/dL)		
	B	β	*p*	B	β	*p*	B	β	*p*	B	β	*p*	B	β	*p*	B	β	*p*
Different BMI levels ^†^																		
BMI < 18.5 (Underweight)																		
Nutrition health behavior	1.079	0.101	0.276	1.139	0.055	0.643	0.819	0.026	0.823	1.089	0.049	0.672	−3.072	−0.126	0.256	10.095	0.146	0.220
Exercise health behavior	−1.130	−0.102	0.237	−1.760	−0.081	0.458	−1.469	−0.044	0.678	−1.990	−0.086	0.423	−0.382	−0.015	0.883	−9.679	−0.134	0.222
18.5 ≤ BMI < 24 (Ideal weight)																		
Nutrition health behavior	0.336	0.024	0.277	0.544	0.016	0.582	−1.411	−0.044	0.090	−0.847	−0.037	0.141	−1.124	−0.039	0.166	0.533	0.003	0.918
Exercise health behavior	−0.947	−0.088	<0.001	−0.737	−0.026	0.315	1.552	0.062	0.012	0.685	0.039	0.116	1.545	0.069	0.006	−12.710	−0.090	0.001
24 ≤ BMI < 27 (Overweight)																		
Nutrition health behavior	0.043	0.003	0.896	−0.151	−0.003	0.915	0.445	0.013	0.662	0.426	0.017	0.553	−0.226	−0.009	0.770	−18.386	−0.076	0.016
Exercise health behavior	−1.067	−0.109	<0.001	−1.447	−0.041	0.191	0.385	0.015	0.626	−0.073	−0.004	0.896	2.290	0.116	<0.001	−3.496	−0.018	0.556
BMI ≥ 27 (Obesity)																		
Nutrition health behavior	0.734	0.037	0.344	0.760	0.013	0.742	−3.158	−0.075	0.050	−1.354	−0.043	0.236	−0.789	−0.031	0.419	−2.672	−0.009	0.831
Exercise health behavior	−2.056	−0.137	<0.001	−2.261	−0.053	0.194	2.541	0.081	0.036	1.076	0.046	0.211	0.731	0.039	0.319	−10.712	−0.046	0.255

Abbreviations: WC, Waist circumference; FBG, Fasting blood glucose; SBP, Systolic blood pressure; DBP, Diastolic blood pressure; TG, Triglyceride; HDL-C, High-density lipoprotein cholesterol; BMI, body mass index. ^‡^ All outcomes of the multiple linear regression analysis are presented in unstandardized coefficients (B) and standardized coefficients (β). Unstandardized coefficient (B) represents the effect of one unit change in the explanatory variable on metabolic parameters levels. For example, in the overweight subgroup, as the exercise score increases one unit, HDL-C increases by 2.290 mg/dL. *p*-value indicates the significance level. ^†^ Each regression was adjusted for gender and age.

## Supplementary Materials

The following are available online at https://www.mdpi.com/1660-4601/16/5/869/s1, Table S1: Crude correlations of metabolic risk factors and exercise and nutrition health behavior ^†^, Table S2: Correlations of metabolic risk factors and exercise and nutrition health behavior for BMI < 18.5 ^†^, Table S3: Correlations of metabolic risk factors and exercise and nutrition health behavior for 18.5 ≤ BMI < 24 ^†^, Table S4: Correlations of metabolic risk factors and exercise and nutrition health behavior for 24 ≤ BMI < 27 ^†^, Table S5: Correlations of metabolic risk factors and exercise and nutrition health behavior for BMI ≥ 27 ^†^.
