# Supplementary material for: Health-Associated Nutrition and Exercise Behaviors in Relation to Metabolic Risk Factors Stratified by Body Mass Index
Source: Int J Environ Res Public Health. 2019 Mar 9;16(5):869. doi: 10.3390/ijerph16050869 (PMC6427460; doi:10.3390/ijerph16050869)
Supplement: Supplementary file 1 [file ijerph-16-00869-s001.pdf]

International Journal of
Environmental Research and Public Health

MDPI

Supplementary Materials: Health-Associated Nutrition and Exercise Behaviors in Relation to Metabolic Risk Factors Stratified by Body Mass Index

Table S1. Crude correlations of metabolic risk factors and exercise and nutrition health behavior [†].

	WC (cm)		FBG (mg/dL)		SBP (mmHg)		DBP (mmHg)		HDL-C (mg/dL)		TG (mg/dL)	
Variables *	r	p	r	p	r	p	r	p	r	p	r	p
**Physical Activity**												
Following a planned exercise program.	−0.065	<0.001	−0.025	0.110	0.085	<0.001	0.112	<0.001	0.112	<0.001	−0.032	0.046
Exercising vigorously for 20 or more minutes at least 3 times per week.	−0.037	0.018	−0.022	0.160	0.100	<0.001	0.112	<0.001	0.106	<0.001	−0.035	0.027
Taking part in light to moderate physical activity.	0.002	0.901	−0.005	0.728	0.075	<0.001	0.075	<0.001	0.039	0.012	−0.031	0.050
Taking part in leisure-time (recreational) physical activities.	−0.031	0.047	−0.042	0.008	0.038	0.017	0.020	0.212	0.040	0.011	−0.045	0.005
Doing stretching exercises at least 3 times per week.	−0.080	<0.001	−0.015	0.354	0.012	0.455	0.033	0.036	0.059	<0.001	−0.021	0.188
Getting exercise during usual daily activities.	−0.082	<0.001	−0.013	0.403	0.046	0.004	0.077	<0.001	0.109	<0.001	−0.035	0.025
Checking pulse rate when exercising.	0.026	0.108	0.033	0.039	0.087	<0.001	0.064	<0.001	0.009	0.587	0.002	0.876
Reaching target heart rate when exercising.	−0.015	0.372	0.009	0.576	0.037	0.022	0.043	0.008	0.015	0.354	−0.034	0.040
**Nutrition**												
Choose a diet low in fat, saturated fat, and cholesterol.	−0.066	<0.001	0.001	0.964	0.020	0.195	0.097	<0.001	0.082	<0.001	−0.012	0.433
Limiting the use of sugars and sweets.	−0.050	0.002	0.020	0.203	0.024	0.134	0.053	0.001	0.074	<0.001	−0.008	0.633
Eating daily servings of bread, cereal, rice and pasta	−0.029	0.073	−0.003	0.866	0.012	0.444	0.020	0.209	0.022	0.183	−0.017	0.293
Eating daily servings of fruit.	−0.006	0.689	0.008	0.603	0.031	0.053	0.092	<0.001	0.059	<0.001	−0.012	0.469
Eating daily servings of vegetables.	−0.056	<0.001	−0.013	0.402	0.016	0.327	0.040	0.011	0.047	0.003	−0.035[*]	0.028
Eating daily servings of milk, yogurt or cheese.	−0.056	0.001	−0.018	0.254	−0.012	0.449	−0.013	0.413	0.057	<0.001	−0.028	0.080
Eating daily servings of meat, poultry, fish, dried beans, eggs and nuts.	−0.006	0.719	−0.001	0.938	−0.024	0.130	−0.026	0.111	−0.008	0.614	−0.005	0.763
Reading labels to identify nutrients.	−0.067	<0.001	0.005	0.744	0.002	0.886	0.055	0.001	0.091	<0.001	−0.052	0.001
Eating breakfast.	−0.050	0.001	−0.026	0.098	0.002	0.884	0.045	0.005	0.083	<0.001	−0.048	0.002

* Abbreviations: WC, Waist circumference, FBG, Fasting blood glucose; SBP, Systolic blood pressure; DBP, Diastolic blood pressure; TG, Triglyceride; HDL-C, High-density lipoprotein cholesterol; BMI, body mass index. [†] Correlations of metabolic risk factors and nutrition and exercise health behavior were examined by bivariate correlation test.

Table S2. Correlations of metabolic risk factors and exercise and nutrition health behavior for BMI < 18.5 [†].

	WC (cm)		FBG (mg/dL)		SBP (mmHg)		DBP (mmHg)		HDL-C (mg/dL)		TG (mg/dL)	
**Variables ***	**r**	**p**	**r**	**p**	**r**	**p**	**r**	**p**	**r**	**p**	**r**	**p**
**Physical Activity**												
Following a planned exercise program.	−0.154	0.109	−0.009	0.923	0.030	0.756	0.019	0.841	0.209	0.028	−0.113	0.238
Exercising vigorously for 20 or more minutes at least 3 times per week.	−0.139	0.147	0.026	0.786	0.112	0.240	0.104	0.279	0.197	0.038	−0.071	0.462
Taking part in light to moderate physical activity.	−0.117	0.222	0.071	0.460	0.080	0.403	−0.001	0.991	−0.061	0.523	−0.031	0.750
Taking part in leisure-time (recreational) physical activities.	−0.082	0.391	0.023	0.813	−0.010	0.919	−0.020	0.835	0.017	0.855	−0.049	0.609
Doing stretching exercises at least 3 times per week.	−0.143	0.133	−0.163	0.085	−0.070	0.464	−0.072	0.448	0.154	0.104	0.002	0.987
Getting exercise during usual daily activities.	−0.125	0.192	0.021	0.826	0.126	0.187	0.177	0.062	0.193*	0.043	−0.020	0.839
Checking pulse rate when exercising.	0.076	0.433	0.191	0.045	0.032	0.742	0.002	0.983	−0.180	0.060	0.080	0.409
Reaching target heart rate when exercising.	0.055	0.583	0.155	0.117	0.029	0.769	0.004	0.971	−0.273	0.005	0.265	0.007
**Nutrition**												
Choose a diet low in fat, saturated fat, and cholesterol.	−0.231	0.015	0.156	0.102	0.054	0.576	0.081	0.397	0.201	0.035	0.071	0.456
Limiting the use of sugars and sweets.	−0.216	0.022	0.060	0.530	−0.010	0.917	0.023	0.809	0.074	0.441	0.230	0.014
Eating daily servings of bread, cereal, rice and pasta	−0.075	0.433	0.055	0.568	0.091	0.347	0.055	0.569	0.014	0.886	0.092	0.342
Eating daily servings of fruit.	−0.116	0.230	0.259	0.006	−0.030	0.755	0.015	0.874	0.061	0.526	0.133	0.168
Eating daily servings of vegetables.	−0.096	0.312	0.066	0.488	−0.014	0.885	0.015	0.874	0.067	0.482	0.074	0.437
Eating daily servings of milk, yogurt or cheese.	−0.056	0.562	0.062	0.521	0.104	0.279	0.100	0.295	−0.033	0.734	−0.152	0.112
Eating daily servings of meat, poultry, fish, dried beans, eggs and nuts.	0.006	0.949	0.006	0.948	0.072	0.837	0.020	0.837	0.100	0.307	0.171	0.080
Reading labels to identify nutrients.	−0.100	0.293	0.112	0.239	0.125	0.190	0.144	0.129	−0.023	0.807	0.124	0.192
Eating breakfast.	−0.096	0.315	0.000	0.994	0.043	0.650	0.081	0.396	0.192	0.043	0.097	0.311

* Abbreviations: WC, Waist circumference, FBG, Fasting blood glucose; SBP, Systolic blood pressure; DBP, Diastolic blood pressure; TG, Triglyceride; HDL-C, High-density lipoprotein cholesterol; BMI, body mass index. [†] Correlations of metabolic risk factors and nutrition and exercise health behavior were examined by bivariate correlation test.

Table S3. Correlations of metabolic risk factors and exercise and nutrition health behavior for 18.5 ≤ BMI < 24 †.

	WC (cm)		FBG (mg/dL)		SBP (mmHg)		DBP (mmHg)		HDL-C (mg/dL)		TG (mg/dL)	
**Variables ***	**r**	**p**	**r**	**p**	**r**	**p**	**r**	**p**	**r**	**p**	**r**	**p**
**Physical Activity**												
Following a planned exercise program.	−0.011	0.648	−0.017	0.461	0.114	<0.001	0.143	<0.001	0.089	<0.001	−0.034	0.149
Exercising vigorously for 20 or more minutes at least 3 times per week.	−0.010	0.657	−.015	0.510	0.123	<0.001	0.134	<0.001	0.090	<0.001	−0.050	0.030
Taking part in light to moderate physical activity.	0.014	0.548	−0.004	0.868	0.060	0.009	0.054	0.020	0.028	0.236	−0.036	0.126
Taking part in leisure-time (recreational) physical activities.	−0.003	0.913	−0.029	0.221	0.047*	0.042	0.024	0.312	0.036	0.123	−0.061	0.009
Doing stretching exercises at least 3 times per week.	−0.076	0.001	−0.001	0.962	0.016	0.488	0.042	0.071	0.045	0.051	−0.031	0.179
Getting exercise during usual daily activities.	−0.062	0.008	0.002	0.918	0.049	0.035	0.081	0.001	0.104	<0.001	−0.015	0.508
Checking pulse rate when exercising.	0.027	0.251	0.030	0.199	0.073	0.002	0.059	0.012	0.016	0.508	−0.007	0.760
Reaching target heart rate when exercising.	0.024	0.329	0.046	0.056	0.071	0.003	0.070	0.004	−0.010	0.682	−0.059	0.015
**Nutrition**												
Choose a diet low in fat, saturated fat, and cholesterol.	−0.027	0.247	−0.001	0.965	0.024	0.298	0.099	<0.001	0.070	0.003	0.009	0.714
Limiting the use of sugars and sweets.	−0.070	0.003	0.025	0.288	0.014	0.550	0.056	0.016	0.093	<0.001	0.018	0.439
Eating daily servings of bread, cereal, rice and pasta	−0.012	0.617	0.005	0.828	0.013	.590	0.035	0.137	−0.012	0.604	0.008	0.733
Eating daily servings of fruit.	0.007	0.774	−0.012	0.614	0.000	0.991	0.067	0.004	0.066	0.005	0.000	0.976
Eating daily servings of vegetables.	−0.046	0.048	−0.024	0.298	0.005	0.838	0.052	0.024	0.048	0.040	−0.029	0.212
Eating daily servings of milk, yogurt or cheese.	−0.104	<0.001	−0.006	0.785	−0.046	0.053	−0.055	0.019	0.081	0.001	−0.052	0.027
Eating daily servings of meat, poultry, fish, dried beans, eggs and nuts.	−0.023	.336	0.025	0.299	−0.045	0.057	−0.031	0.188	−0.019	0.417	−0.002	0.946
Reading labels to identify nutrients.	−0.102	<0.001	−0.019	0.408	−0.008	0.740	0.038	0.099	0.099	<0.001	−0.034	0.144
Eating breakfast.	−0.029	0.217	0.007	0.760	0.018	0.450	0.057	0.015	0.073	0.002	−0.021	0.364

* Abbreviations: WC, Waist circumference, FBG, Fasting blood glucose; SBP, Systolic blood pressure; DBP, Diastolic blood pressure; TG, Triglyceride; HDL-C, High-density lipoprotein cholesterol; BMI, body mass index. † Correlations of metabolic risk factors and nutrition and exercise health behavior were examined by bivariate correlation test.

Table S4. Correlations of metabolic risk factors and exercise and nutrition health behavior for 24 ≤ BMI < 27 [†].

	WC (cm)		FBG (mg/dL)		SBP (mmHg)		DBP (mmHg)		HDL-C (mg/dL)		TG (mg/dL)	
Variables *	r	p	r	p	r	p	r	p	r	p	r	p
**Physical Activity**												
Following a planned exercise program.	−0.051	0.071	−0.018	0.515	0.074	0.009	0.112	<0.001	0.129	<0.001	0.003	0.910
Exercising vigorously for 20 or more minutes at least 3 times per week.	−0.070	0.012	−0.016	0.578	0.082	0.003	0.100	<0.001	0.142	<0.001	−0.015	0.588
Taking part in light to moderate physical activity.	0.037	0.191	0.000	0.982	0.104	<0.001	0.106	<0.001	.076	0.007	−0.006	0.829
Taking part in leisure-time (recreational) physical activities.	−0.049	0.076	−0.031	0.264	0.046	0.096	0.028	0.324	0.064	0.022	−0.044	0.116
Doing stretching exercises at least 3 times per week.	−0.033	0.245	−0.007	0.803	0.008	0.774	0.034	0.229	0.062	0.026	0.000	0.978
Getting exercise during usual daily activities.	0.009	0.756	0.000	0.998	0.085	0.002	0.124	<0.001	0.078	0.005	−0.003	0.926
Checking pulse rate when exercising.	−0.026	0.353	0.023	0.419	0.065	0.020	0.030	0.285	0.037	0.192	−0.009	0.741
Reaching target heart rate when exercising.	−0.027	0.343	−0.038	0.184	−0.003	0.914	0.000	0.999	0.050	0.080	−0.023	0.422
**Nutrition**												
Choose a diet low in fat, saturated fat, and cholesterol.	−0.026	0.355	0.020	0.473	0.059	0.034	0.135	<0.001	0.061	0.029	−0.002	0.951
Limiting the use of sugars and sweets.	−0.022	0.429	0.015	0.595	0.052	0.062	0.070	0.012	0.064	0.023	−0.034	0.228
Eating daily servings of bread, cereal, rice and pasta	−0.020	0.489	0.005	0.868	0.033	0.241	0.021	0.455	0.027	0.341	−0.021	0.471
Eating daily servings of fruit.	−0.005	0.857	0.010	0.729	0.068	0.016	0.108	<0.001	0.059	0.036	0.037	0.193
Eating daily servings of vegetables.	−0.033	0.241	−0.009	0.756	0.065	0.021	0.053	0.056	0.016	0.559	−0.039	0.166
Eating daily servings of milk, yogurt or cheese.	−0.035	0.216	0.004	0.898	0.042	0.141	0.060	0.033	0.044	0.118	−0.056	0.047
Eating daily servings of meat, poultry, fish, dried beans, eggs and nuts.	−0.015	0.586	−0.057	0.045	−0.005	0.861	−0.010	0.735	0.038	0.175	−0.014	0.634
Reading labels to identify nutrients.	−0.051	0.067	0.024	0.398	0.022	.432	0.081	0.004	0.079	0.005	−0.061	0.029
Eating breakfast.	0.021	0.458	−0.033	0.242	0.036	0.197	0.080	0.004	0.048	0.084	−0.037	0.192

* Abbreviations: WC, Waist circumference, FBG, Fasting blood glucose; SBP, Systolic blood pressure; DBP, Diastolic blood pressure; TG, Triglyceride; HDL-C, High-density lipoprotein cholesterol; BMI, body mass index. [†] Correlations of metabolic risk factors and nutrition and exercise health behavior were examined by bivariate correlation test.

Table S5. Correlations of metabolic risk factors and exercise and nutrition health behavior for BMI ≥ 27 [†].

	WC (cm)		FBG (mg/dL)		SBP (mmHg)		DBP (mmHg)		HDL-C (mg/dL)		TG (mg/dL)	
Variables *	r	p	r	p	r	p	r	p	r	p	r	p
**Physical Activity**												
Following a planned exercise program.	−0.063	0.086	−0.019	0.601	0.141	<0.001	0.152	<0.001	0.074	0.042	−0.026	0.471
Exercising vigorously for 20 or more minutes at least 3 times per week.	−0.069	0.057	−0.043	0.239	0.116	0.001	.126	<0.001	0.108	0.003	−0.037	0.312
Taking part in light to moderate physical activity.	−0.053	0.144	−0.020	0.573	0.080	0.027	0.101	0.005	0.053	0.145	−0.067	0.063
Taking part in leisure-time (recreational) physical activities.	−0.071	0.048	−0.082	0.024	0.035	0.329	0.026	0.478	0.006	0.868	−0.015	0.675
Doing stretching exercises at least 3 times per week.	−0.061	0.091	−0.008	0.830	0.098	0.007	0.101	0.005	−0.016	0.660	0.022	0.551
Getting exercise during usual daily activities.	−0.101	0.005	−0.023	0.528	0.063	0.080	.073	0.043	0.077*	0.034	−0.058	0.110
Checking pulse rate when exercising.	0.015	0.686	0.031	0.392	0.139	<0.001	0.117	0.001	0.031	0.394	0.003	0.935
Reaching target heart rate when exercising.	−0.008	0.824	0.019	0.616	0.063	0.090	.094	0.012	0.036	0.329	−0.002	0.955
**Nutrition**												
Choose a diet low in fat, saturated fat, and cholesterol.	0.000	0.993	0.008	0.822	0.049	0.179	0.147	<0.001	0.023	0.526	0.000	0.990
Limiting the use of sugars and sweets.	0.005	0.880	0.038	0.295	0.052	0.149	0.070	0.053	0.003	0.936	0.001	0.982
Eating daily servings of bread, cereal, rice and pasta	0.018	0.634	−0.012	0.739	0.014	0.713	0.023	0.534	0.049	0.188	−0.025	0.500
Eating daily servings of fruit.	−0.024	0.513	0.031	*0.397*	0.065	0.077	0.152	<0.001	0.063	0.085	0.001	0.971
Eating daily servings of vegetables.	−0.005	0.881	0.023	0.534	0.033	0.359	0.057	.114	0.014	0.707	0.000	0.986
Eating daily servings of milk, yogurt or cheese.	0.000	0.997	−0.063	0.083	−0.009	.815	−0.028	.451	−0.011	0.767	0.079	0.031
Eating daily servings of meat, poultry, fish, dried beans, eggs and nuts.	0.001	0.986	0.030	0.416	−0.037	0.317	−0.057	0.125	−0.086	0.019	−0.010	0.786
Reading labels to identify nutrients.	0.001	0.983	0.036	0.321	0.022	0.547	0.085	0.020	0.074	0.040	−0.051	0.157
Eating breakfast.	−0.027	0.454	−0.040	0.267	−0.014	0.708	0.034	0.348	0.079	0.030	−0.065	0.074

* Abbreviations: WC, Waist circumference, FBG, Fasting blood glucose; SBP, Systolic blood pressure; DBP, Diastolic blood pressure; TG, Triglyceride; HDL-C, High-density lipoprotein cholesterol; BMI, body mass index. [†] Correlations of metabolic risk factors and nutrition and exercise health behavior were examined by bivariate correlation test.
